# Reafference and the origin of the self in early nervous system evolution

**DOI:** 10.1098/rstb.2019.0764

**Published:** 2021-03-29

**Authors:** Gáspár Jékely, Peter Godfrey-Smith, Fred Keijzer

**Affiliations:** ^1^ Living Systems Institute, University of Exeter, Stocker Road, Exeter, EX4 4QD, UK; ^2^ School of History and Philosophy of Science, University of Sydney, New South Wales 2006, Australia; ^3^ Department of Theoretical Philosophy, University of Groningen, Groningen, The Netherlands

**Keywords:** reafference, early animal evolution, self, sensing, non-bilaterian, corollary discharge

## Abstract

Discussions of the function of early nervous systems usually focus on a causal flow from sensors to effectors, by which an animal coordinates its actions with exogenous changes in its environment. We propose, instead, that much early sensing was *reafferent*; it was responsive to the consequences of the animal's own actions. We distinguish two general categories of reafference—translocational and deformational—and use these to survey the distribution of several often-neglected forms of sensing, including gravity sensing, flow sensing and proprioception. We discuss sensing of these kinds in sponges, ctenophores, placozoans, cnidarians and bilaterians. Reafference is ubiquitous, as ongoing action, especially whole-body motility, will almost inevitably influence the senses. Corollary discharge—a pathway or circuit by which an animal tracks its own actions and their reafferent consequences—is not a necessary feature of reafferent sensing but a later-evolving mechanism. We also argue for the importance of reafferent sensing to the evolution of the *body-self*, a form of organization that enables an animal to sense and act as a single unit.

This article is part of the theme issue ‘Basal cognition: multicellularity, neurons and the cognitive lens’.

## 1. Introduction

Work on early nervous system evolution is generally shaped by the assumption that the main function of a nervous system is to control behaviour [1,2]. This task includes both adjusting action to the circumstances with the aid of the senses, and also the internal coordination of behaviour itself—shaping the micro-acts of parts of the body into the macro-acts of the whole [3–5]. Here, we look specifically at the side of neural evolution that involves behaviour and its relation to sensing; we offer a reconceptualization of this aspect of neural evolution. The early functions of nervous systems probably also included the control of physiological processes and ontogeny [5], but these aspects are not considered here.

It has often been natural to explore this topic by considering ancient forms of sensing—chemotaxis, phototaxis, various forms of touch—and locating them in a causal flow in which external conditions are sensed and lead to a behavioural response. A tradition of work on more neurally complex animals, including arthropods and vertebrates, has argued for a different view of these relationships between sensing and action, one that makes central the concept of *reafference*: the effects of action on what is sensed [6] (see box 1 for a glossary of terms). Extending and redirecting these ideas, we develop the concept of reafference through the general principle that self-initiated action evokes sensory change, and then apply these ideas to early nervous system evolution. We show how reafference manifests itself in a number of senses—gravisensing, flow sensing, sensing associated with stretch—in non-bilaterian animals and simpler bilaterians. Through these examples, we also illustrate how the body's layout and form and its sensory systems have coevolved to use reafferent sensing. Reafference thus provides a unifying concept for neural and body-plan evolution. These considerations also shed new light on the origin of a ‘self’ in animal evolution, which we formalize in the concept of the *body-self*.

Box 1. **Glossary of terms**

*Reafference*: any effect on an organism's sensory mechanisms that is due to the organism's own actions

*Reafference principle*: self-initiated action evokes sensory effects that are correlated with these actions and, therefore, can be predicted and used

*Exafference*: any effect on an organism's sensory mechanisms that is due to external conditions or events

*Corollary discharge*: an internal pathway by which an animal tracks its own actions and their predicted reafferent consequences

*Statocyst*: specialized sensory cells or organs that track the motion of some part affected by gravity as the animal changes orientation

*Proprioception*: sensing of deformations, stresses and other mechanical changes within the body

*Tensegrity*: a design principle that is followed to build structures from rods under compression with attached cables imposing the compression

*Deformational v. translocational reafference*: reafference relating to body deformations as contrasted with reafference involving movement in relation to a medium or field

*Body-self*: a form of organization including motility, reafferent sensing and morphology enabling the organism to act as a single unit

*Ctenophores*: also called comb jellies, are gelatinous marine invertebrates that represent one of the earliest branching metazoan groups

*Placozoa*: disc-shaped millimetre-sized marine animals that glide upon surfaces by cilia

*Choanocyte chamber*: internal cavity in the aquiferous system of sponges with choanocytes that act as pumping and filtering units

*Lateral line*: a canal system with sensory ciliated cells that allows aquatic animals to detect fluid motion relative to the body

## 2. The reafference principle

The concept of reafference was introduced by von Holst & Mittelstaedt ([6]; see also [7]) as part of a rival to a prevailing view of neural activity based on reflex arcs, with their simple flow from sensory stimulus to response. Von Holst and Mittelstaedt [6] argued for ‘a complete reversal of the usual way of looking at the system’, one that starts with action and inquires into the consequences of those actions on the senses—those consequences are reafference. Part of this reversal was a model in which animals continually establish and maintain states of ‘equilibrium’ by filtering their raw sensory input with ‘efference copies’ that register their own actions; animals then refer the ‘residual’ of what is sensed to higher control centres as input that is indicative of externally caused events, or exafference.

This focus on efference copy and Sperry's [8] related notion of a corollary discharge tend to cast reafference as a disturbance of perception—and hence a problem—for which specific neural circuitry has evolved to compensate [9,10]. Here, we employ reafference in a more general way, one that stresses the importance of self-induced action as a central ingredient for perception. One way of casting this idea is in terms of a control loop where behaviour acts as a device that controls perceptual input [11–13]. A variety of related ideas are proposed in embodied approaches to cognition where action is held central to perception [14,15] and the role of the body is stressed in creating and shaping reafferent relations [16,17]. The way in which forward motion produces an optic flow across the visual field [18] is another example of a wider usage of reafference.

Here, we offer a view of reafference that goes beyond its original restricted use, but differs also from many of the broader theoretical claims that others have made for it. We do not hold that, in the light of reafference, perception itself becomes a form of action [15], or, conversely, that the function of action is to control perception [12] or to make it as predictable as possible [19]. From a biological point of view, action has many roles other than this. We do not use reafference to assimilate perception to action, or vice versa, though we do seek to reconceptualize their relationship.

Our discussion is guided, first, by what we will call the *reafference principle*: that self-initiated action evokes sensory effects that are correlated with these actions and, therefore, can be predicted and used. We understand reafference itself as any effect on an organism's sensory mechanisms that is due to the animal's own actions. A single sensory mechanism may on different occasions respond to reafferent or exafferent events; reafference is a feature of sensory episodes, not mechanisms themselves. The paradigm examples involve motion of the body, but even a sessile animal can act with reafferent consequences, as when a filter-feeding animal generates a feeding current by motile cilia.

Second, reafference provides an opportunity, a resource, that can be exploited by animals. Many organisms use motion to elicit stimuli from the environment that would not otherwise arise. A bacterial example would be the way in which *Escherichia coli* and other bacteria use motility to assess the presence of a chemical gradient [20,21], while Gibson's ecological account of perception [18] articulated this idea in detail for animal vision. Self-initiated motion provides a stream of clues about environmental configurations. Reafference is not restricted to external motion but also applies to self-induced changes in body postures and feedback on imposed force as in active touch [22].

Third, given that self-initiated activities tend to have predictable consequences, reafference constitutes feedback concerning such predictions [13]. In this way, reafference provides a means by which organisms can evaluate these predictions and modify the activity involved. This need not involve a nervous system. For example, in sponges, sensory cilia keep track of the flow produced within the body and can signal when this flow ceases [23]. In animals with nervous systems, a *corollary discharge* is a more sophisticated mechanism that compensates for predicted sensory changes by registering the particular action underway at a time. With Crapse & Sommer [9], we use ‘corollary discharge’ rather than ‘efference copy’ to refer to this broad category.

Fourth, we differentiate between two forms of reafference, those relating to body deformations and those without shape changes but involving translocation or other movement (e.g. rotation) in relation to a medium or field (water, air, visual environment, magnetic or gravitational field). During *deformational* reafference, changes in the shape of the body lead to sensing, such as during proprioception. During *translocational* reafference, self-initiated motions induce an interaction with the environment with consequences for sensing (e.g. various flows). The impact of movement on statocysts and vestibular changes also belong in this category. The two categories are not mutually exclusive, and come together, for example, during active touch.

Most of the senses will be affected by the animal's own actions, and will hence give rise both to reafferent and to exafferent sensory episodes; as seen below in the case of gravisensing, a sensory event may be due to self-caused motion or the action of waves. ‘Reafferent’ and ‘exafferent’ as defined here apply to episodes, classified according to their causes, rather than to mechanisms. However, we can also envisage a purely exafferent sensory mechanism—one that never produces reafferent episodes—such as an ambient light-sensing mechanism in a sessile organism, used to tune circadian metabolism. In that case, a stimulus guides an adaptive response, but the response has no effect on subsequent sensory events of that kind.

**Figure 1.**
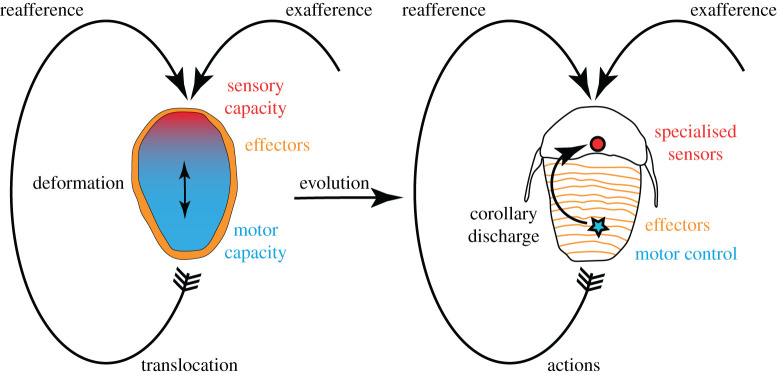
Schematic of a basic and an evolutionary more advanced form of reafference. The scheme on the left represents an early animal with deformational reafference with an internal reciprocal influence between effector and sensory events. The schematic on the right depicts a more evolved animal with specialized sensors and effectors and corollary discharge mechanism. Deformational reafference is the sensory effect of a physical contraction of the body. Corollary discharge refers to a neuronal signal to filter reafferent sensing during action. (Online version in colour.)

Here, we use the idea of reafference to cast light on early neural evolution and early animal life. Rather than a setting where specific neural circuits are being added to an already active animal, the question is how lumps of cells—with specific cell characteristics—evolved into organisms with many different cell types, a highly differentiated morphology and physiological organization, and sophisticated capacities for action and perception (figure 1).

## 3. The body-self

Another aim of this paper is to introduce and use a concept which we call the *body-self.* The term ‘self’ has many senses, some that involve complex thought and conscious experience, and some that are intended to be less demanding. Damasio [24] introduced the idea of a *proto-self*, to refer to a collection of brain devices that represent and maintain various states of the body in a range suitable for survival. His proposal, and others [25], can be seen as attempts to describe simpler precursors of the conscious human self. However, views like these still assume fairly high levels of biological organization, including the existence of a complex brain. Our concept is intended to pick out a more basic kind of self, one arising earlier in evolution, but one that is not trivially equivalent to the concept of an organism or physical object.

An organism has, or embodies, a body-self if it has a particular form of organization. That form of organization includes motility (of the whole or parts) and sensing, where action and sensing are tied together through reafference. The body-self then encompasses the devices and their activities that enable reafferent coupling between the animal's own actions and sensing. The body-self can thus include sensors and effectors, their activity or actions, and also the form of the body influencing reafferent coupling. In this view, brains, if they are present, are not the sole locus or even the centre of this self, but a part of the body that is characterized by this self. The body-self enables the organism to sense and act as a single unit, and thus a self that separates itself from the rest of the world.

A body-self has a non-arbitrary differentiation from its environment (though with some vagueness of boundaries); it marks itself off as a unit by the organization of its action, sensing and physical form. A body-self, when present, becomes a platform for further evolutionary innovation, including new kinds of sensing that draw on the prior demarcation of self from environment.

We apply the concept of the body-self to multicellular animal bodies. Unicellular organisms also have a form of self-hood, but it is simpler. They are divided from the environment by a single membrane and their activity is coordinated within that boundary (e.g. by ionic currents or second messengers). In a multicellular context, self-hood has to be reestablished by the coordination of parts and through reafferent sensorimotor loops. We acknowledge that reafference can also be relevant to describe the behaviour of single-celled eukaryotes but we do not discuss unicellular examples here (for an extensive discussion of single-celled behaviour, see [26]).

In the next sections, we discuss some forms of sensing and action that have a close relationship to the evolutionary emergence of the body-self in animals. These ‘self-forming’ sensory capacities feature reafference of various kinds, and also have a plausible role in early animal evolution.

## 4. Reafferent sensing and body-to-environment translocation

This section begins discussion of a range of examples of reafferent sensing in animals, especially non-bilaterian animals, and some other cases with relevance to the early history of animals. Early animals had limited bodily resources and simple nervous systems, when these were present at all. Our argument is that a significant and widespread feature of early animal evolution was putting available resources to work in handling and using reafferent connections between sensing and acting. No extant animals, even simple ones, can be assumed to resemble ancestral forms, but by means of a survey of present-day animals, we hope to show that a range of sensory capacities that plausibly feature in early animal evolution are capacities in which reafference plays an important role. Animals were building their ability to accommodate and use reafference as they were evolving their ability to sense and act. We begin our survey with cases of translocational reafference, one of two categories distinguished above.

### (a) Gravity sensing

Once an animal is actively moving in any three-dimensional medium, it will tend to reorient in relation to Earth's gravity vector. If orientation is important (e.g. during vertical migration in the water column), a need will arise to control it by sensing and responding to orientation. Insofar as orientational changes to the gravity vector are produced by active motion, their sensory effects are reafferent in our sense.

The ability to sense the orientation of one's own body relative to Earth's gravity vector is present in many animals. Such active gravity sensing coupled to effector systems can lead to reorientation movements, maintaining or restoring a desired body orientation (as opposed to passive gravi-orientation). Gravity sensing relies on specialized cells or organs called statocysts in many animals [27]. Statocysts have a cavity containing small concretions or statoliths. When the animal changes its orientation relative to the gravity field, the statoliths move in the cavity and stimulate mechanosensory cells lining the cavity. The signal for the statocyst is generated by the tilt of the body and can lead to a response (e.g. the animal ‘righting’ itself). Such tilt may come about by self-generated movements or external forces (e.g. water turbulence). When actively induced tilt has sensory consequences, this qualifies as reafference. Reafferent gravisensing then contrasts both with exafferent gravisensing (in response, for example, to turbulence or waves), and with passive gravi-orientation, where the body acts as a buoy, as a consequence of the distribution of mass in its physical layout. Given that self-initiated activity almost inevitably leads to changes in the orientation of the body, reafferent gravity sensing will be useful as a means to compensatory reorientation.

**Figure 2.**
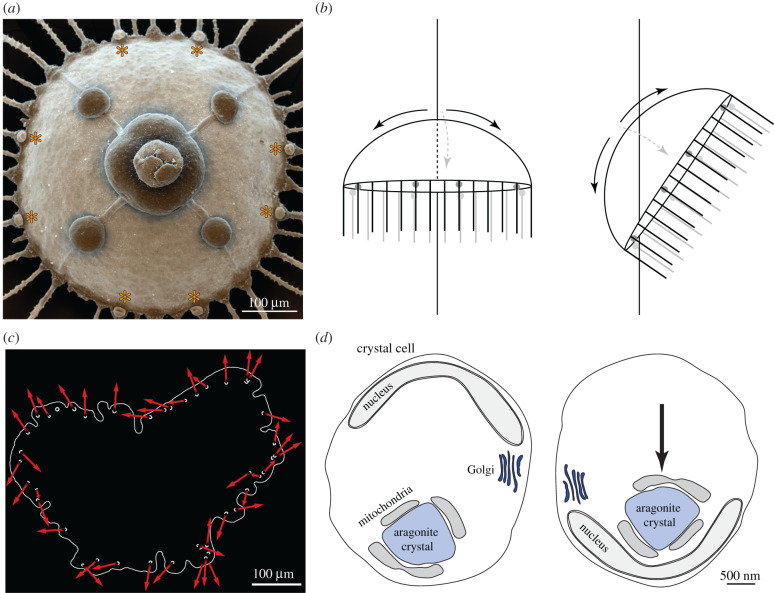
Statocysts in a jellyfish and the placozoan *T. adhaerens*. (*a*) Scanning electron micrograph of a jellyfish, seen from below, showing the tentacles, statocysts (marked by asterisks), manubrium and gonads. Image by Jürgen Berger. (*b*) Schematic of a jellyfish changing its orientation. The statocysts are positioned around the circumference of the umbrella, and hence can signal body tilt in all directions. (*c*) Schematic of a *Trichoplax* and its crystal cells. Arrows show the orientation of the cup-shaped nuclei. Image from Mayorova *et al.* [31]. (*d*) Schematic of a crystal cell in different orientations relative to the gravity vector. The aragonite crystal in the cell moves relative to the nucleus. This movement likely induces a signal in the cell. After Mayorova *et al.* [31]. (Online version in colour.)

The widespread use of statocysts across non-bilaterian animals suggests that they may have evolved early in animal evolution. In cnidarian medusae, there are several statocysts at regular intervals at the base of the tentacles [28–30] (figure 2). Ctenophores have a single statocyst in their aboral sense organ [32]. The statolith in this organ is attached to four groups of ciliated cells [33], one in each quadrant of the animal. Upon a change in body posture, the statolith presses the cilia and changes their beating frequency. This change propagates to the locomotor ciliary comb plates to reorient the body. The sensory excitation is graded with the beating frequency of the cilia changing as a function of statolith load [34].

In the placozoan *Trichoplax adhaerens*, an animal lacking true muscles or a nervous system, there are crystal cells at regular intervals at the perimeter of the animal, each containing a 1–3 µm diameter aragonite crystal (figure 2). Upon changes in body orientation relative to gravity, the crystal shifts downwards within the cell. Animals lacking crystal cells are unable to move against gravity on a tilted plane [31]. Statocysts are also present in several bilaterians, including molluscs [35–37] and some annelids [38,39]. In gastropods, the pattern of responding hair cells in the statocyst is thought to allow the animal to determine its spatial orientation with respect to gravity [40].

There are also other types of gravity sensors. In flies, for example, the Johnston's organ is the gravity-sensing organ and it detects movements of the antenna [41,42]. A further example is the system of cercal gravity receptors in crickets with club-shaped sensilla that work like pendulums [43–45].

In gravity sensing and gravity responses, the reafferent coupling between actions and senses can only work if certain conditions are met. (i) The gravisensors need to be organized such that they can differentially signal the tilt of the body along all its cardinal axes, (ii) the sensory signals need to be graded in proportion to the magnitude of a tilt [34,46], and (iii) the statocysts need to control effector organs (muscles or cilia) such that the animal can right itself (gravi-orientation) and move up or down (gravitaxis). In such cases, we can speak of a gravisensory module of the body-self, encompassing the devices and their activities that deal with gravity in an active manner through reafferent coupling. As gravisensory modules evolve, this will affect nervous system organization because the body form affects the placement of sensors which influences nervous anatomy.

Elemental gravisensory systems can serve as a platform for the evolution of more complex circuitry and behaviours. One possible elaboration is to evolve the ability to switch the sign of gravitaxis depending on multimodal input deriving from other exterosensors. Such multimodal regulation of statocysts occurs, for example, in the pond snail *Lymnaea stagnalis* where low oxygen concentration switches gravitaxis from positive to negative [46]. In ctenophores, mechanosensory stimuli induce a switch in the sign of gravitaxis [34]. Another elaboration is to differentiate between reorientation caused by self-movement and reorientation due to external perturbations. Such differentiation is not essential for gravireception to function, but it could allow more elaborate motor control and would require a form of corollary discharge. In more advanced cases, statocysts can be recruited to the control of quite complex behaviours. In the marine planktonic pteropod mollusc, *Clione limacina*, the statocysts are involved in generating a complex swimming trajectory during hunting [36]. In evolution, statocyst networks can be elaborated, receive input other than orientational cues and even evolve intrinsic dynamics to guide complex behaviour through their motor connections.

### (b) Flow sensing

Just as active motion induces changes in relation to the Earth's gravitational field, in aquatic organisms, it also induces flow. Flow sensors, widespread in aquatic animals, generally consist of one or more mechanosensory cells which have a sensory cilium deflectable by flow [27]. The cilium can be surrounded by microvilli, forming a mechanosensory apparatus where deflections are transduced into cellular signals by mechanosensory ion channels.

Through the example of flow sensation, we will illustrate how filter feeders and active swimmers use reafferent flow sensing and how reafferent sensing facilitates detection of exafferent causes of sensory change. We also show how reafferent feedback depends on the size and shape of the body and discuss some physical principles such as buoyancy, Reynolds numbers and flow fields, and how these impinge on reafference and corollary discharge.

Sensing changes in water flow can be relevant for both swimming and sessile organisms. Sessile or planktonic filter-feeding animals including sponges, ascidians, anthozoans and many other animals can generate feeding currents by cilia or muscular appendages (e.g. copepods) [47]. If the animal can sense this self-generated flow, it is readily enabled to detect deformations in the flow field caused by clogging or approaching objects such as predators distorting the flow field.

**Figure 3.**
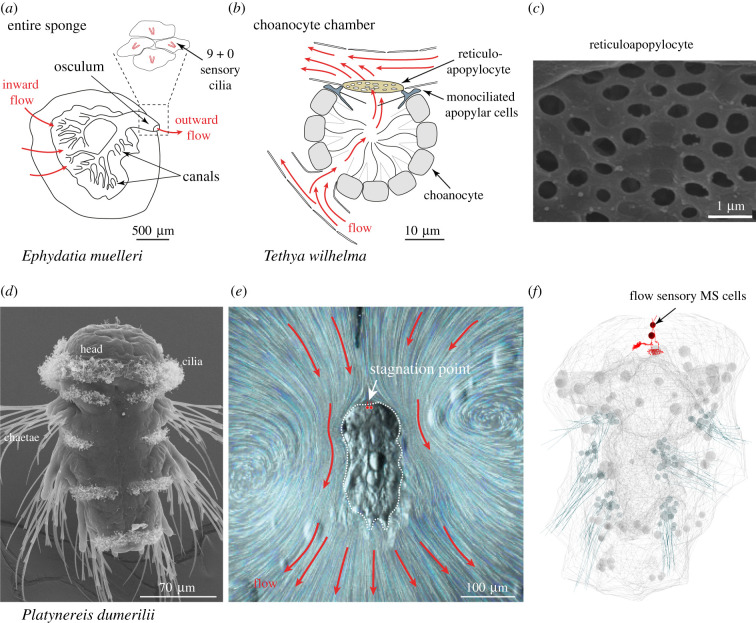
Flow-sensory systems in sponges and an annelid larva. (*a*) Schematic of the freshwater demosponge *Ephydatia muelleri*, after Leys & Meech [48]. Inset shows a slice of the internal wall of the osculum containing putative flow-sensory cells with 9 + 0 sensory cilia. (*b*) Schematic of a choanocyte chamber in *T. wilhelma*, after Hammel & Nickel [49]. (*c*) Scanning electron microscopy image of a reticuloapopylocyte in *T. wilhelma* from Hammel & Nickel [49]. (*d*) Scanning electron microscopy image of a 3-day-old *P. dumerilii* larva. (*e*) Flow field around a tethered *P. dumerilii* larva, beating with its locomotor cilia. Flow rates are very low at the stagnation point indicated by the white arrow. Red dots mark the position of the flow-sensory MS1 and MS2 cells. The flow was visualized by fluorescent microbeads. Image from Bezares-Calderón *et al.* [50]. (*f*) Morphology of two flow-sensory MS neurons in the 3-day-old *P. dumerilii* larva as reconstructed by serial electron microscopy [50]. (Online version in colour.)

In sponges, putative flow-sensory cells in the osculum may sense a reduction in feeding flow from the choanocyte chambers due to clogging, initiating contractions to expel waste [23] (figure 3). Sponges also have an active control over the volume of water they filter and this depends on external flow [51]. In the demosponge *Tethya wilhelma*, a putative flow-regulating reticular cell type, the reticuloapopylocyte, has been described. This cell is situated at the excurrent pore of the filtering choanocyte chambers and has a fenestrated morphology with openings of variable diameter [49]. How self-induced flow, environmental flow and flow sensing interact to regulate flow rates is unclear, but it is possible that sponges have the ability to integrate information about the state of their canal system and environmental flows.

Self-propelled ciliated larvae of metazoans can orient in flow fields [52] and can have flow-sensory cells. In larvae of the annelid *Platynereis dumerilii*, flow sensors (MS cells) are positioned at the most anterior tip of the head [50] (figure 3). In the flow field around the anterior tip of *Platynereis*, there is a stagnation point, where the local velocity of the fluid is zero. Such stagnation points are a general feature of the flow fields around self-propelled microswimmers (e.g. [53,54]). In *Platynereis* larvae, some of the MS cells are located at this stagnation point. This suggests that these cells will not be exposed to self-induced flow and may respond to external shear flow more efficiently. The filtering-out of reafferent stimulation, in a way akin to corollary discharge, is in this case achieved by the precise placement of sensors in a position defined by the hydrodynamics of the self-propelled body.

The shape and even the buoyancy of the body of small planktonic organisms can have various and non-intuitive hydrodynamic effects on the flow fields around a swimming body, the swimming trajectories and body orientation [55]. Larvae of the sand dollar, an echinoderm, are transported upwards or downwards in vertical flows depending on larval stage and morphology [56]. Detailed flow-field measurements around a swimming *Volvox carteri*—a colonial green alga—revealed that flows are dominated by a component due to gravity and the negative buoyancy of the colony [54]. Similar detailed measurements have not been carried out for planktonic animals, but many metazoan larvae are in the size range of *Volvox* (*R* ∼ 200 µm). If a planktonic organism has flow-sensory systems, these various hydrodynamic effects could thus dictate the optimal positioning of those sensors. This is one of many ways in which the physics of the body could feedback onto nervous system evolution.

A more advanced flow-sensing system is the lateral line in fish, tadpoles, lampreys [57] and possibly amphioxus [58]. During swimming, the lateral line experiences reafferent signals and the magnitude of these signals is tuned by modulatory efferent neurons. In tethered fish during fictive swimming, the spontaneous spiking rate of the lateral line afferent neurons decreases, in correlation with motoneuron activity [59].

The reafferent signal in swimming organisms also depends on the size of the body and size-related hydrodynamic effects. At the scale of ciliary microswimmers including animal larvae and other small plankton, viscous forces dominate over inertial forces (low Reynolds numbers). By contrast, larger animals like fish operate at higher Reynolds numbers where inertia is more important. A fish after a swim bout will glide in the water, without motor activity. During gliding, reafferent signals can still activate the lateral line. The corollary discharge can persist during the glide phase [59], suppressing reafferent signals even without a motor action. This is a good example to illustrate that in a corollary discharge system, it is not sufficient to have a simple ‘subtraction’ of the motor command itself, but the system needs to predict the consequences of the motor action, given the nature of the body and environmental setting. In the fish example, inertia, given the body and its milieu, is one physical aspect that will influence how an action plays out.

Similar principles could be applied to the evolution of visual systems and optic flow sensing. The first eyes in animal evolution likely already relied on reafferent sensing. The simplest eyes are non-visual phototactic eyespots that rely on the helical rotation of the swimming body to scan the light field [60–62]. For organisms with more complex visual eyes, self-induced optical flow provides an important mechanism to orient themselves with respect to the environment. The changes in visual texture, signalled by the light falling on an array of photoreceptors, provide the animal with information about objects, pathways to traverse and imminent collisions [18,63–65]. In a way that is comparable to the forward point of stasis in *Platynereis* larvae, the direction of movement is simply signalled by the point in the visual array from which all other points diverge. In addition, in many cases, corollary discharge mechanisms are present. In the fly compound eye, for example, voluntary turns are associated with an efference copy that suppresses the response to the turn in the visual cells [66]. A similar suppression of movement signal happens during saccades in the primate eye [67].

## 5. Reafferent sensing and body deformation

We now address forms of reafferent sensing that keep track of changes in the body itself. Although the term ‘reafference’ has been most often used for effects of action on exterosensors, the distinction between self-caused and other-caused sensory events (reafference and exafference) is also available in the case of interoception. Sensing body deformation involves a wide array of proprioceptors, for which we use Lissmann's long-standing definition: ‘Sense organs capable of registering continuous deformation (changes in length) and stress (tensions, decompressions) in the body, which can arise from the animal's own movements or may be due to its weight or other external mechanical forces' (cited by Mill [68, p. xvi]). In this context, the physical characteristics of the animal body are central, as well as the various ways in which it can be actively deformed by its own activity. Here, we will first describe the notion of tensegrity structures as a way to integrate a wide array of processes and forms of organization that are involved in body deformation and reafference, including biochemical, biomechanical, physiological and cytological processes, as well as the overall organization of animal body shape. This will provide the background to discuss reafferent sensing in touch and epithelial stretch, and in muscle proprioception.

### (a) Animal bodies as tensegrity structures

At heart, the animal body is a soft deformable structure, built up from epithelia folded and expanded during development. In contrast with plants and fungi, animal cells have no rigid cell wall and the combined cells lack rigidity that has only secondarily, and not in all cases, been reinforced with specialized structures, including sponge spicules and a variety of internal and external skeletons made up from various materials (shell, bone, chitin, etc.). These skeletons notwithstanding, the animal organization remains one that can be dynamically deformed on short notice by contractions of muscle cells, or precursors thereof, that can both deform and stabilize body shape [69,70] and be used to initiate bodily movements.

The animal body shape is a dynamic feature even when it is outwardly unchanging. A useful concept here is *tensegrity* or *tensional integrity*. Tensegrity is a general design principle that is followed to build structures from rods under compression with attached cables imposing the compression. The integrity of the structure arises from a combination of rigid and elastic components combined under tension. This form of organization also applies to the animal body. Here, a skeleton constitutes the rigid parts that oppose compression, while muscle and tendons (mostly) constitute the flexible component that, by means of tensile forces, binds the skeleton together [22].

For early animal evolution, three differences with the original tensegrity concept are relevant. First, the tensile components can change length by muscle contraction and relaxation, making the tensegrity structure capable of dynamic and reversible changes. Second, early cases did not have hard skeletons, so the opposing force for a muscle system derives instead from more diffuse hydrostatic skeletons that, like water-filled balloons, provide a flexible but incompressible mass [71]. Third, the dynamically changing mechanical forces involved in these animal tensegrity structures themselves constitute signals that travel across large parts of the body—like using a connecting rope to ring a faraway bell—and influence biochemical processes at the cellular level. For example, mechanical connections between extracellular fibres and the cytoskeletons of individual cells modify the latter [72–74] and stretch-activated ion channels change their bioelectrical properties under mechanical stress [75,76]. At larger scales of aggregation, proprioceptive sensors add to the neural modulation of such changes [68].

Dynamic tensegrity structures constitute an active and very flexible motile organization, which requires suitable (and complex) coordinative control to maintain body shape [74,77] and organize behaviour [3,5,22] as also can be witnessed in work on soft robotics [78–80].

The sensitivity of cellular processes to the dynamically changing pattern of mechanical forces across the tensegrity structure makes reafference an intrinsic ingredient of this organization. Self-generated forces imposed on the structure will influence proprioceptive sensors both at a cellular and at a multicellular scale [81]. The importance of force-dependent molecular switches that react to developmental tissue deformations has been well established [73–77]. Here, we address behavioural examples involving body deformation where reafferent sensing plays various roles.

### (b) Proprioception

All animals are able to actively change their body shape through actomyosin-mediated contractions. Ctenophores, cnidarians and bilaterians use muscles for shape changes. In placozoans, ultra-fast epithelial contractility underlies shape changes [82]. Some sponges also undergo coordinated contractions, involving various contractile cell types in their canal system and outer surface [83].

**Figure 4.**
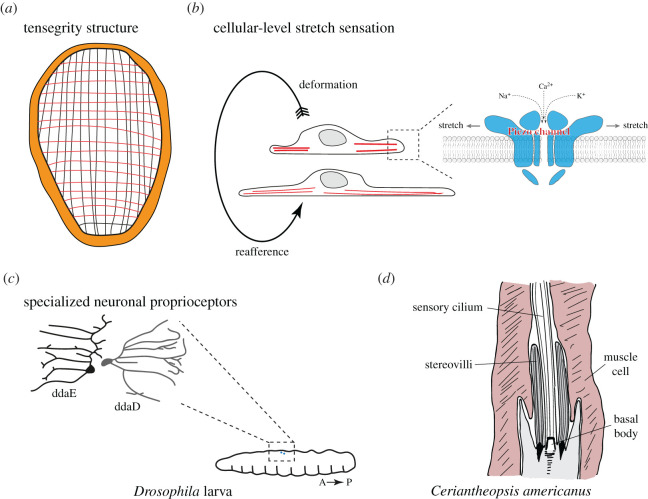
Stretch sensation and proprioreception. (*a*) Schematic of a hypothetical early animal with orthogonal muscle fibres forming a tensegrity structure. (*b*) Cellular-level stretch sensation by mechanosensory channels. Upon deformation or contraction, stretch-sensitive channels (e.g. Piezo) can open, leading to an internal reafferent feedback. (*c*) Specialized neuronal proprioreceptors in the fly larva. The ddaE and ddaD neurons have sensory dendrites that deform during larval crawling. ddaE is more active during forward locomotion, while ddaD activates during backward locomotion. Sketch after He *et al.* [84]. (*d*) Sketch of a putative cnidarian proprioceptor in the cerianthid *C. americanus*, after Bezares-Calderón *et al.* and Peteya [27,85]. The sensory dendrite including the cilium and the stereovilli are embedded in a muscle cell. Muscle contraction may provide proprioceptive feedback. (Online version in colour.)

Cellular sensors responsive to deformation or contraction-induced stresses might evolve as an intrinsic ingredient of such dynamic shape changes. When the contractile effectors and connected tissues are stretched or compressed, this could induce mechanosensory currents, providing the basis for reafferent signals (figure 4). Compared to body-to-environment translocation, the presence and the nature of such proprioceptive reafference are more difficult to establish. We first give an overview of bilaterian cases of proprioception, before discussing the possibility of its presence in non-bilaterian animals and when in animal evolution it might have appeared.

Proprioceptors sensing muscle stretch are widespread and well studied in bilaterians. In crawling *Drosophila* larvae, different proprioceptors sense body wall deformations either during muscle contractions or extension [86]. There are also different subtypes of proprioceptive neurons that either are more active during forward or during backward locomotion [84] (figure 4). Proprioception in the fly is dependent on the pan-metazoan transmembrane channel *Tmc* [87] that may sense the membrane curvature in proprioceptor dendrites [84]. The proprioceptors provide feedback about body position by synaptic connections to premotor neurons [88].

Adult *Drosophila* incorporate a broad variety of mechanoreceptors, many of which act as proprioceptors [89]. For example, chordotonal organs are found on exoskeletal joints and between joints within limb and body segments [90]. Campaniform sensilla are oval domes on the exoskeleton with a thin cuticle covering a sensory dendrite. They sense mechanical stresses that bend the exoskeleton. Stretch-sensitive receptors also occur in connective tissue or linked to muscle. The mechanosensitive ion channels TRP-N [91] and Piezo-like channels [92] are involved in proprioception. A detailed study of leg proprioceptors revealed subgroups encoding leg position, movement direction and vibration frequency [93]. Crabs have similar systems sensing muscle stretch and leg position [94].

Proprioception is also well understood in the nematode *Caenorhabditis elegans* [95]. One of the stretch-sensitive proprioceptive neurons, DVA, expresses a transient receptor potential (TRP) channel TRP-4 (a homologue of TRP-N/NompC) and regulates locomotion behaviour. *Trp-4* mutant worms show abnormal body bending and posture [96]. SMDD is another proprioceptive neuron that is activated during head steering locomotion to modulate the curvature of forward motion. Two TRP channels, *trp-1* and *trp-2*, are necessary for head proprioception [97]. In addition, the body wall muscles themselves are also mechanosensitive [98]. Movement not only affects neuronal activity through proprioception in *C. elegans*, but also through corollary discharge. Head motor commands during head movements are encoded by the RIA interneurons that are reciprocally connected to head motoneurons. Movements are encoded on a subcellular scale in RIA neurons through compartmentalized calcium dynamics [99,100].

In vertebrates, proprioceptors sensing stretch are found in muscle spindles and Golgi tendon organs [101]. Mechanosensitivity in these sensory cells is dependent on the ion channels Piezo2 and Tentonin3 (TTN3) [102–104]. The proprioceptive cells feed back onto motor neurons in the spinal cord [101]. Defects in proprioception can lead to abnormal gait and a loss of coordination of body movements [105]. In fish, mechanosensory feedback during movement is provided by spinal cord sensory neurons (Rohon–Beard neurons and Kolmer–Agduhr cells). The mechanosensitivity of the Kolmer–Agduhr cells depends on the polycystin channel Pkd2l1 [106,107].

The evidence supporting the presence of muscle or stretch proprioceptors in non-bilaterians is at best fragmentary. Cnidarians and ctenophores can have relatively complex muscular behaviours [32,108], and some form of proprioception may be present to ensure coordinated behaviour. An electron microscopic study by Peteya [85] reported putative proprioceptive cells in the cerianthid cnidarian *Ceriantheopsis americanus* (figure 4). These cells have a ciliated sensory apparatus that runs parallel to the long axis of the animal's body. The sensory cells have both afferent and efferent synapses suggesting a feedback system. The efferent synapses derive from the motoneurons innervating the body wall and may modulate the sensory cell's sensitivity during muscle contractions [85]. Similar cells with their cilia closely associated with epidermal circular muscles are found in the tentacles of the cubozoan jellyfish *Carybdea marsupialis* [109].

Placozoans undergo substantial and rapid changes in body shape by fast epithelial contractions [82]. It remains unclear whether these shape changes induce any stretch-dependent monitoring of body shape.

Some sponges can also actively change their body shape by contraction and extension [51,110–112]. A well-known case is the ‘sneeze’ of the freshwater sponge *E. muelleri*. This sponge uses peristaltic-like contractions to expel clumps of waste material from its water canal system, suggesting ‘that control over a hydrostatic skeleton evolved prior to the origin of nerves and true muscle’ [83, p. 3736; 113]. The fine tuning of the canal diameter may be linked to flow sensation at the excurrent canals, as discussed above. Flow sensation of the contractile state of the canal system may be considered as an indirect form of proprioceptive feedback.

A further interesting example of putative proprioceptive systems comes from studies of sponge and coral larval settlement. Whalan *et al*. observed that sponge larvae preferentially settle in holes with a size that matches the size of the larva. This selective settlement guided by surface microtopography may be achieved by mechanosensation [114].

The ancestral presence of various mechanosensory ion channels in animals indicates that some form of mechanosensing was present at the origin of the animals. At least initially, this may only have been a form of cellular or tissue-level stretch sensing and without specialized mechanosensory cells. One class of ancient ion channels present in animals and many protists is the Piezo mechanosensory channels [115]. These channels are required for mechanically induced currents in cells and could serve as cell-autonomous stretch sensors. Piezo has also been identified in *Trichoplax* and may induce currents upon shape changes [116].

The mechanosensory TRP-N family (first described as NompC in *Drosophila* [117]) is also ancient and is present in cnidarians, ctenophores and placozoans but not poriferans [87,118]. TRP-N mediates many mechanosensory functions such as the control of body movement and perception of touch in nematodes [119] and in flies [91]. Besides TRP-N, up to six TRP channel families date back to the origin of animals or before [87].

Given the ubiquity of mechanotransduction channels, it may be that mechanosensation represents one of the oldest sensory processes that evolved in animals, directly connected to contraction-based motility [76].

## 6. Reafference and the evolution of animal bodies and nervous systems

Along with new species and new traits, evolution occasionally produces new kinds of living units—new kinds of selves. The nature of such a new form can include the layout and materials of the body, capacities for acting and sensing, and systems of coordination and control, such as nervous systems and others. The animal body-self is one such form of organization, resulting from evolutionary change in all these areas. The existence of a body-self is a matter of degree. In its paradigm cases, a body-self is unified by neural control, reafferent sensing and a suitable morphology, all of which facilitate action at a multicellular level. Non-neural animals can have a partial body-self of this kind, as can physically connected colonial forms where self-hood is distributed between a collective and its constituent zooids. During evolution, the mechanisms enabling unified sensing and action, with accompanying morphologies, became more elaborate, giving rise to body-selves of different varieties. We sketch here some possible pathways in early animal and neural evolution that relate to the body-self and draw on the ideas in earlier sections of this paper.

Unicellular organisms are compact enough to behave as units in quite complex ways without large-scale coordination of parts. The origin of animals produced larger units, composed of many cells, that were often invested in a lifestyle that puts a premium on coordinated action on their new spatial scale. Cell–cell signalling, eventually including nervous systems, became the basis of this coordination.

A plausible starting point for the animal body-self can be found in collections of cells that came to act as dynamically changing soft-bodied tensegrity structures. The shape changes of such structures depend on a self-imposed and self-maintained interplay of compressive and tensile forces that, in turn, can modify cellular signalling in various ways. An animal body is, therefore, not merely a collection of cells, but an integrated unit tied together by mechanical forces. When appropriately coordinated, the collective becomes a unit capable of doing mechanical work—changing its shape and moving—and also becomes a platform for various sensory devices.

In almost any unit that can both sense and act, alongside the theoretically familiar causal paths from sensing to action, there will be pathways *from* action *to* the senses. Reafference is an almost inevitable consequence of the combination of acting and sensing. Reafference brings with it both ambiguities in sensory input and opportunities to actively probe environments. It is a feature of sensing even before the evolution of nervous systems. The evolution of sensory systems will have been affected by the near-inevitability of reafference from early stages. This phenomenon will be particularly marked in the case of the sometimes neglected forms of sensing we have discussed in this paper: gravisensing, flow sensing, stretch sensing and proprioception. Sensitivity to the consequences of action of this kind may also establish, through its shaping of sensitivity on a multicellular scale, paths to new forms of exterosensing. These paths eventually yield forms of sensing in which reafference and exafference combine tightly together, in actively moving animals, including lateral line sensing in fish and active vision as discussed by Gibson [63].

Non-neural animals are restricted to limited coordination and agency. Their bodies, while materially unified, are not tied together as selves in the same way that a neuralian animal is. Despite this, their sensing will include reafference to some degree. Nervous systems then bring with them new possibilities for integration. As well as the familiar ways that a nervous system integrates control, the expansion of agency that nervous systems make possible can shape the form of the body itself. Work in *Hydra* has revealed several non-overlapping neural networks responsible for particular behaviours [120]. In most cases, these networks are each spread throughout the entire body, but remain distinct from each other; they do not form a single connected ‘nerve net’. Though the networks are distinct, their interaction within a soft body may generate not only behavioural sequences but also aspects of the body's form. Dupre & Yuste suggest that the *Hydra* morphology may result from the ‘push–pull’ action of two opposed ectodermal networks, constituting a soft-bodied tensegrity organization.

Even unconnected neural networks within a single behaving body will be linked by reafference. Proprioception may also be present. However, a point can be made here about the different roles, within cnidarian lifestyles, of polyp and medusa forms. The polyp body plan is thought to be the ancestral form in cnidarian evolution, with the medusa appearing later. A medusa actively swimming in the water column engages in more organized, integrated behaviours. A polyp, in contrast, might get by with less integrated control, as seen in *Hydra*. Sensory systems are also more elaborate in the medusa form, with gravisensing (discussed above) and, in some cases, significant visual ability [121]. A related evolutionary pathway—perhaps overlapping at early stages—may exist in ctenophores. Here, too, a plausible scenario has a sessile polyp-like form as ancestral, perhaps existing before a branching that produced cnidarians and ctenophores, still with polyp-like bodies [122,123]. From there, both lineages evolved a more active medusa-like form. Ctenophores use cilia rather than body contraction for swimming, though muscle is employed in steering. In both cases, the more active, motile form brings with it a more integrated variant of the body-self. Sophisticated reafferent sensory systems such as statocysts also seem to have evolved independently in the medusoid form in ctenophores and cnidarians. The large degree of parallel evolution between ctenophores and cnidarians plus bilaterians—as evidenced by molecular comparisons [124]—may partly be due to their independent conquest of the pelagic zone.

The evolution of the bilaterian body plan brings with it a further expansion of behavioural repertoire, with new possibilities of mobility and manipulation. Common across a range of animals whose genealogies coalesce only in the protostome-deuterostome common ancestor (nematodes, arthropods, vertebrates and others), nervous system activity includes global or brain-wide dynamic patterns that are associated with particular actions [125]. In some cases, the expansive spread of these patterns makes problematic any simple distinction between ‘sensory’ and ‘motor’ areas in an animal. These are action-directed patterns that can also be modulated by impinging external events.

Across a similarly wide range of bilaterians, reafference is addressed and mobilized with corollary discharge mechanisms. Reafference becomes not just a standing fact about the relations between sensing and acting, but something whose presence shapes neural architecture, which now includes circuitry that modulates the processing of sensory signals according to what the animal is currently doing.

At present, corollary discharge mechanisms of this kind are not known in non-bilaterian animals. This has two possible explanations. First, it may be that the utility of these mechanisms is tied to the breadth of a behavioural repertoire. When an action is produced continually or routinely (a swimming motion, perhaps), its reafferent consequences can be handled implicitly, without a neural circuit indicating which action is being produced at a particular time. Alternatively, such mechanisms may be present, though not yet observed; perhaps even routinely produced motions require registration of ongoing actions as part of regulatory feedback. If so, we hypothesized that if corollary discharge mechanisms are present in cnidarians, their likely location is in association with advanced sensory mechanisms in medusoid forms, such as statocysts and cubozoan eyes [121].

The evolution of corollary discharge mechanisms, in response either to an expansion of behavioural repertoire or the demands of fine control, has further consequences. An animal with this organization handles sensory events in a way that includes an active neuronal marking of the distinction between *self* and *other*. Its organization embodies a self in a richer sense. Organisms without a neuronal corollary discharge may already have the ability to distinguish self and other through intrinsic differences in sensory events (e.g. between active contraction or being squeezed). We mentioned Damasio's notion of the ‘proto-self’ as a candidate for a kind of implicit self-concept that is prior to a fully fledged, reflective human sense of self. As noted, Damasio's proto-self is dependent on extensive neural organization and internal sensing. Before the proto-self arose, animals were not just objects with various sensory and effective adaptations collected together. They were integrated in a way that gave them an earlier kind of self-hood. We introduced the concept of the body-self to describe the devices and activities that enable reafferent coupling between the animal's own actions and sensing, together with the body's own layout, all of which enable the organism to sense and act as a single unit. As we have argued throughout the paper, to get going as an animal, you need a body-self, with its utilization of reafference, pretty early. The body-self then provided a platform for further stages in animal evolution, including the evolution of complex nervous systems, and more elaborate and explicit forms of the self.

## Data accessibility

This article has no additional data.
